# Translating Mechanism of Regulatory Action of Tolerogenic Dendritic Cells to Monitoring Endpoints in Clinical Trials

**DOI:** 10.3389/fimmu.2017.01598

**Published:** 2017-11-22

**Authors:** Jessica S. Suwandi, Tatjana Nikolic, Bart O. Roep

**Affiliations:** ^1^Department of Immunohematology and Blood Transfusion, Leiden University Medical Center, Leiden, Netherlands; ^2^Department of Diabetes Immunology, Diabetes & Metabolism Research Institute, Beckman Research Institute, City of Hope, Duarte, CA, United States

**Keywords:** tolerogenic dendritic cells, monitoring endpoints, clinical trials, autoimmune diseases, regulatory action, antigen specific, regulatory T cells, immune metabolism

## Abstract

Tolerogenic dendritic cells (tolDCs) have reached patients with autoimmune and inflammatory disease, at least in clinical trials. The safety of tolDCs as intervention therapy has been established, but the capacity to modulate autoimmune response *in vivo* remains to be demonstrated. Studies have revealed a diversity of regulatory mechanisms that tolDCs may employ *in vivo*. These mechanisms differ between various types of modulated tolDC. The most often foreseen action of tolDCs is through regulatory polarization of naïve T cells or activation of existing regulatory T cells, which should ultimately diminish autoimmune inflammation. Yet, selection of a target autoantigen remains critical to expedite tissue specific tolerance induction, while measuring immune modulation incited by tolDCs *in vivo* provides a great challenge. We will discuss the regulatory action of different types of tolDCs and the possible methods to monitor immunological efficacy endpoints for the next generation clinical trials.

## Introduction

The regulatory properties of dendritic cells (DCs) have been subject of study throughout the last decade (1–5). The ability of DCs to orchestrate the immune system makes them interesting candidates for therapeutic application. In autoimmune diseases where the physiological state of self-tolerance is lost, tolerogenic dendritic cells (tolDCs) could aid in restoring the immunological balance. Several modulating actors have proved to induce DCs with stable regulatory capacity and researchers have since developed clinical grade tolDCs suitable for clinical trials (6–8). Phase I clinical trials with tolDCs are ongoing or have been completed in patients with type 1 diabetes (T1D), rheumatoid arthritis (RA), Crohn’s disease and multiple sclerosis proving tolDC vaccination safe and well tolerated, encouraging next generation trials to verify the therapeutic efficacy (9–13). While disease amelioration is the goal in the long run, immunological changes may be detectable more promptly and understanding the regulatory mode of action of tolDCs is essential to define immunological efficacy endpoints.

Variation in the methods used for culture makes comparison of tolDCs difficult, may therefore lead to diversity and inconsistency when comparing results from clinical trials evaluating different tolDCs in different diseases or conditions. In addition, tolDCs with desired therapeutic efficacy have not been identified yet. Current actions such as that of Action to Focus and Accelerate Cell-based Tolerance-inducing Therapies (http://www.afactt.eu) have generated minimum information models to report and interpret data on the quality and preclinical efficacy of tolDCs (14, 15). This may enable the comparison of treatment effects of tolDCs generated with other methods. In this review, we consider regulatory actions of tolDCs and discuss the methods to monitor these *in vivo* as immunological efficacy endpoints for future clinical trials, whether they are described as a common feature or shown only for a certain type of tolDC. Using similar immunomonitoring strategies in different trials could also help answering the question whether the variation in the culture methods translates into variable functional properties.

## Phenotypical Characteristics and Cytokine Profile of tolDCs—Mediators for Tolerogenic Function

Several approaches have been tested to induce maturation resistant tolDCs *in vitro* (2, 7, 16, 17). Common features of tolDCs presumed to mediate tolerogenic functions include low antigen presentation capacity, reduced co-stimulatory signals, expression of inhibitory molecules and an anti-inflammatory cytokine profile. Co-stimulatory signals such as CD80, CD86, and CD40 in addition to antigenic stimulation are key to adequate T cell activation and absence thereof leads to unresponsiveness, i.e., anergy and activation of regulatory T cells (Tregs) (18, 19). The balance between pro- and anti-inflammatory cytokines IL-12 and IL-10 is important for tolerance. IL-12 is central in the induction of T helper 1 cells (Th1) and high IFN-γ production. By contrast, IL-10 reduces the antigen-presenting function of DCs, inhibiting Th1 responses (20). Furthermore, presence of IL-10 is a requisite for the induction of a subset of Tregs (type 1 Treg), while Forkhead box P3 (Foxp3) demethylation is dispensable, rather than a condition *sine qua non* (21–23).

An overview of phenotype and functions of clinically applied tolDCs is provided in Table 1, showing variations of the abovementioned common traits as well as unique features that may initiate regulation through distinctive mechanisms. Most tolDCs show reduced expression of co-stimulatory molecules and HLA-DR, while expressing inhibitory molecule PD-L1 (8, 10, 24, 25). tolDCs treated with antisense oligonucleotides against co-stimulatory molecules CD40, CD80, and CD86 (antisense tolDC), and NF-kB inhibitor (NF-kB tolDC) demonstrate low TNF and IL-10 production (10, 26). By contrast, tolDCs induced with combined dexamethasone and vitamin A or vitamin D3 show high production of IL-10 (8, 24, 25, 27). Gene and protein expression data revealed CD52 as candidate marker specifically for VitD3-Dex-modulated tolDC (28) and MERTK was identified in Dex-VitA tolDCs as a specific molecule involved in the negative regulation of T cell activation (29), yet these markers remain to be validated in other tolDCs. Although efforts have been made to find molecules underlying tolDC function, common regulators of tolerogenicity have not been found (28, 30).

**Table 1:** Characteristics of clinically applied tolDCs.

Modulator tolDC	Disease	Phenotype	Cytokine production	Inhibition of T cell proliferation	Induction of Treg	Regulation of B cells	Reference
Antisense oligonucleotides targeting CD40, CD80, and CD86	T1D	↓ CD40, CD80, and CD86 (mouse)	↓ IL-12p70, NO, TNF-α (mouse) No IL-10 or IL-4 (human *in vitro*)	n.a.	Increased CD4+ CD25+ (in NOD mice)	n.a.	(26)
				n.a.	No increase in CD4+ CD25+ Foxp3+	Upregulation of B220+ CD11c−CD19+ lymphocytes with *in vitro* regulatory capacity	(11)
NF-kB signal inhibitor (Bay11-7082)	RA	↑ HLA-DR ↑ CD86, CD40 ↓ CD80	↓ TNF ↓ IL-10 ↓ IL-6	Reduced Ag specific proliferation (mouse draining lymphnode)	CD4+ CD25+ Foxp3+ Treg	Isotype switch IgG2b to IgG1 and IgA (mouse)	(24)
		↑ PD-L1 ↓ PD-L2		Reduced IL-6 response to one of the vaccinated antigens Reduction of CD4+ CD25+ CD127+ T-eff cells	n.a.	Reduced anti-CCP IgA/IgG levels	(10)
Dex/Vit A/cytokine-mix (IL-1β, IL-6, TNF-α, PGE2)	Crohn’s	↓ HLA-DR ↓ CD80, CD83 ↑ CD86	↑ IL-10 No IL-12 No IL-23	Reduced Ag-specific proliferation and induction of anergy	n.a.	n.a.	(8)
		MERTK		n.a.	Significant increase CD4+ CD25++ Foxp3 Tregs	n.a.	(12)
Dex/VitD3 (Dex day 3, Dex + VitD3 day 6)	RA and IA	↓ HLA-DR ↓ CD40, CD80, CD83, CD86 ↑ CD14	↑ IL-10 ↓ IL-12 ↓ IL-1β, IL-6, IL-23, TNF-α	Anergy in memory T cells Reduced proliferation of autologous T cells (with recall antigen)	IL-10-producing Tregs CD4+ IL-10+ CD25−Foxp3− (Tr-1 like Tregs)	Increase of CD19+ IL-10+ Bregs	(31)
							(24)
		↑ TLR-2 ↓ PD-L1 ↑ PD-L2		n.a.	No increase in CD4+ Foxp3+ Tregs	n.a.	(9)
VitD3/Dex (VitD3 day 0, VitD3 + Dex Day 3)	T1D	↓ HLA-DR ↓ CD40,CD80, CD83, CD86 ↑ CD14 ↑ PD-L1	↑ IL-10 ↓ IL-12	Reduced proliferation of CD4 and CD8 T cells	CD25+ Foxp3+ CD127− Tregs CD25+ Foxp3− Tregs Tr-1 like Tregs Granzyme B+ Tregs CTLA-4+ IL-10+ Tregs Tregs with inverse TCR docking	n.a.	(23, 25, 28)
		↑ CD52 ↑ ILT-3		n.a.	n.a.	n.a.	n.a.

*Evidence from preclinical studies (light blue) and clinical studies (dark blue)*.

*↓, Low expression/secretion; ↑, high expression/secretion; T1D, type 1 diabetes; RA, rheumatoid arthritis; IA, inflammatory arthritis; Crohn’s, Crohn’s disease; tolDC, tolerogenic dendritic cell; Tregs, regulatory T cells; CTLA-4, cytotoxic T lymphocyte-associated protein 4; Foxp3, Forkhead box P3*.

While the knowledge about ligands and soluble mediators help us understand how tolDCs shape immune response and may be utilized as clinical release criteria for *in vitro* generated tolDCs, none of them have proved to be unique to serve as a biomarker of tolDCs *in vivo*, while their efficacy to achieve therapeutic efficacy remains to be confirmed.

## Hyporesponsiveness of Effector CD4 and CD8 T Cells

A common trait of tolDCs is the suppression of effector T cells (Table 1) (2). tolDCs inhibit T cell proliferation either directly by inducing anergy or apoptosis, or through the induction of Tregs. Death receptor ligands such as PD-L1 function as direct negative regulator of T cell response. tolDCs treated with VitD3 delete T cells antigen specifically with co-ligation of PD-1 (32). Another mechanism through which Dex-VitA tolDCs inhibit T cell proliferation is through MERTK. MERTK is a family of TAM tyrosine kinase receptors and directly inhibits T cell activation through competition of PROS1 on the surface of T cells, which drives autocrine proliferation (29). Furthermore, VitD3-Dex tolDCs inhibit naïve CD8 T cell proliferation and induce anergy in memory CD8 T cells. However, this effect is countered by cytotoxic killing of tolDCs presenting CD8 epitopes (33). Whether other tolDCs similarly affect CD8 T cells, needs to be verified.

Altogether, tolDCs are capable of inhibiting T cell proliferation through different mechanisms. This common feature is ideal to utilize as efficacy endpoint in clinical trials. *In vivo* alterations of CD4+ T cell responses can be determined with a lymphocyte stimulation test (LST) and enzyme-linked immunosorbent spot assay (34), which quantifies antigen-specific T cell proliferation and cytokine secretion in human peripheral blood mononuclear cells. The LST was proven valid in predicting graft survival in pancreatic islet transplantation, since increase of proliferation was associated with a rapid failure of islet grafts (35). Effects on T cell populations could be further assessed through quantification of effector CD4 T helper subsets (Th1, Th2, and Th17) and CD8 T cells by flow cytometry. Moreover, using quantum dot nanotechnology (Qdot) it is possible to detect and quantify autoreactive CD8 T cells (36). *In vivo* signs of T cell modulation were already observed in the NF-kB tolDC trial by a reduction of CD4+ CD25+ CD127+ effector T cells (10).

*In vivo*, tolDCs could alter different T cell subsets with the potential to influence overall disease outcome as affected subsets may have specific pathophysiological relevance for a particular autoimmune disease. The inflammatory reaction in RA and Crohn’s disease is mediated by T helper 1 and 17 (Th1 and Th17) cells secreting pro-inflammatory cytokines IFN-γ, IL-17, and IL-22 (37, 38). By contrast, autoreactive CD4 T helper cells contribute to T1D pathogenesis but cytotoxic CD8 T cells are the main offenders, destroying the insulin producing beta cells (39–41). Therefore, harmonizing assays and following changes in multiple T cell subsets in response to tolDC treatment could enable comparison and correlation to clinical outcomes in different trials.

## Induction of Tregs

Perhaps the most important and diverse mechanism of tolDCs is the induction of Tregs, which has been demonstrated *in vitro* and *in vivo* (17). These induced Tregs are suspected to suppress pathogenic autoimmune processes by effector T and B cells involved in a multitude of autoimmune diseases. So far, several Treg populations have been described, and tolDCs can induce or activate various Tregs depending on the DC modulating agent. Naturally produced thymic Tregs (nTregs) are defined using the high and stable expression of transcription factor Foxp3 and represent the best described Treg subset next to CD4+ Foxp3− type 1 Tregs (Tr-1) producing high IL-10. *In vitro*, NF-kB tolDCs and Dex-DCs promote CD4+ CD25+ Foxp3+ Tregs, while tolDCs modulated by Dex plus VitD3 also induced Tr-1 like Tregs (23, 24, 31). This is in line with the thought that Tr-1 Treg induction is dependent on IL-10 production by tolDC (Table 1) (21, 22). Membrane bound TNF and PD-L1 are other factors involved in the induction of antigen-specific Tregs and may contribute to the capacity of VitD3-Dex tolDCs to induce heterogeneous Treg subsets which suppress through distinct mechanisms such as killing of monocytes and inhibition of naïve or effector T cell proliferation (23, 42, 43). Indeed, tolDCs generated by VitD3-Dex induce at least three different types of Tregs (23). Whether these features are shared by Tregs induced by different types of tolDCs, and whether the variety of Tregs induced by tolDCs extends to other types remains to be investigated. Induction of Tr-1 like Tregs seems preferred over nTreg induction, since the antigen specificity of the latter is undefined. nTregs may therefore suppress any effector T cell response, including those against cancer, whereas Tr-1 cells with defined specificity will exclusively exert their action when their cognate target of choice (e.g., islet autoantigen) is recognized.

Similar to determining effector T cell responses, the quantification and qualification of Tregs in patients in relation to tolDC treatment is an essential tool in all trials. Indeed, an upregulation of CD4+ CD25+ Foxp3+ Tregs was observed in humans injected with Dex-VitA tolDCs (12). Most studies were limited to measuring circulating Foxp3+ Tregs which possibly underestimates the therapeutic effect, since Tr-1 Tregs induced by IL-10 producing tolDCs need not express Foxp3 (21–23). Another problem with simply looking at Foxp3 expression is that this transcription regulator is also transiently expressed in activated T cells, thus CD4+ CD25+ Foxp3+ cells represent a mixture of both Tregs and activated effector T cells (44, 45). Lastly, an observed increase of CD4+ CD25+ Foxp3+ cells could be indicative of an expansion of dedicated nTregs or newly induced Tregs from naïve T cells in the periphery (45, 46). Additional markers such as ICOS, PD-1 and cytotoxic T lymphocyte-associated protein 4 (CTLA-4) could help us describe suppressive cells in response to tolDC action. Yet, due to a lack of a common marker for all Tregs, measuring the suppressive capacity of T cells in a suppression assay remains the only valid method to determine whether Tregs are present (47, 48).

## Infectious Tolerance and Linked Suppression

It becomes increasingly clear that the interaction between Tregs and DCs is bidirectional since DCs induce Tregs, which in turn impact DC development reducing co-stimulatory ligands and stimulation of suppressive molecules (49, 50). CTLA-4 expression on Tregs modulates DCs by scavenging the co-stimulatory ligands on DCs through the process of trans-endocytosis (51). Tregs induced by VitD3-Dex tolDCs stimulate the expression of inhibitory B7-H3 and ICOS ligand (i.e., B7-H2) on inflammatory DCs upon cognate interaction, which thereafter induced IL-10 producing T cells with other antigen specificities causing infectious tolerance (50). Another molecule described on modified DCs is B7-H4, which is up-regulated under influence of IL-10 secretion by CD4+ CD25+ Tregs (52). Hence, tolDC can exert infectious tolerance through the capacity of Tregs to induce linked suppression and potentially modulate other DCs *in vivo* to acquire tolerogenic phenotype and function. In this way, induced Tregs can augment the suppressive capacity of tolDCs by transferring regulatory properties to other inflammatory DCs. So far, this complementary action is proven in VitD3-Dex tolDCs but is yet to be validated in other, such as antisense and NF-kB tolDCs. *In vivo*, analysis of DCs acquiring the expression of inhibitory molecules from the B7 family (B7-H2, B7-H3, and B7-H4) or a spreading of tolerance to antigens other than that carried by tolDC-vaccine may be an additional lead to monitoring of tolerance induction in the trials and create legacy of tissue specific immune regulation beyond the lifetime of the injected tolDCs.

## Importance of Antigen-Specific Tolerance Induction

The ultimate goal of tolDC therapy is the induction of targeted tolerance, thereby impeding autoimmune inflammation in the affected lesion. Addition of one or more target antigen(s) will guide tolDCs to address effector cells which is desirable to induce disease-relevant immunomodulation. For this purpose, established disease-associated autoantigens are necessary. This may be a straight-forward approach in the case of T1D and multiple sclerosis where tissue specific antigens are identified as suitable targets (53–57). Since tolDCs induce Tregs that act through linked suppression, regulation will not be limited to the antigen to which the Tregs were generated, but spread to all other specificities presented by residency DCs in the lesion or draining lymph nodes. However, in some autoimmune diseases, specific autoimmunity-inducing antigens are unknown or associated antigens are not tissue specific. Citrullinated antigens and dead-cell-related epitopes associated with RA and systemic lupus erythematodes, respectively are present throughout the body, which obscures the desire to induce specific tolerance (3, 58). Antigens involved in Crohn’s disease also remain unidentified despite great efforts (12, 37). In the latter case, application of tolDCs will rely on the migratory capacity of tolDCs to the pathogenic lesion and local uptake of proteins and presentation in tolerogenic context. In whichever way it may be achieved, the antigen specificity of tolerance induction is essential to avoid general immune suppression and should be closely monitored, for example by measuring the proliferative response against pathogens included in the childhood immunization program.

## Suppression of B Cells

A rarely studied effect of tolDCs is the regulation of B cells, as suggested by preliminary clinical data, but the clinical relevance of such B cell modulation *in vivo* needs to be confirmed. Patients with RA treated with citrullinated peptide loaded NF-kB tolDCs showed reduced anti-CCP IgA/IgG levels, which correlated with clinical improvement (10). Similarly, a significant reduction of antigen-specific autoantibodies was observed in another clinical trial with tolDCs in RA patients (13). The mechanism through which tolDCs regulate B cells is still undiscovered. DCs play an important role in the function of B cells through transferring antigens to naïve B cells and initiation of antigen-specific antibody responses. In addition, DCs provide B cells with isotype-switch signals and promote B cell proliferation and survival through CD40 (59, 60). It is plausible that tolDCs lack the capacity to stimulate B cells resulting in reduced activity of plasma cells or regulate B cell activity indirectly by inducing Tregs. Inhibition of B cell function may not be equally important in all autoimmune diseases as the role of B cells in the pathophysiology of T1D is largely elusive, and islet specific antibody titration does not correlate with disease progression (55, 61, 62). Yet, regardless of whether B cells are pathogenic, Bregs may still prove valuable in disease modulation (62, 63).

More recent data show evidence of tolDC involvement of Breg induction (11). B cells with suppressive activity (Bregs) have been described in the past, but their biology is just beginning to unravel. The so-called Bregs regulate through promotion of Treg development and suppression of effector CD4 and CD8 T cells (64). The phenotype of Bregs could be characterized by the expression of various surface markers (CD19, CD21, and CD23) and the expression of IL-10. Recipients of antisense tolDC vaccination showed an increase of IL-10 producing Bregs in peripheral blood, these Bregs inhibited allogeneic T cell proliferation *in vitro* independent of Tregs (11, 65). Dex-VitD3 tolDCs increased a population of CD19+ IL-10+ Bregs *in vitro* (24). The underlying mechanism of Breg induction is largely unknown, and IL-10 may be involved. More specifically in the case of antisense tolDC, the antisense oligonucleotide mixture may stimulate expression of CD40L and IL-7 on tolDCs and drive Breg induction (24, 65).

Dependent on the pathophysiology of the disease in question, quantification of B cell populations or measuring of disease-specific antibody titers could be relevant. The potential role of tolDCs in Breg induction should be further explored in other types of tolDCs and may proof relevant as additional player with regulatory property.

## Potential Metabolic Effects of tolDCs

Gene expression data and proteomics have revealed considerable changes in metabolic pathways in tolDCs induced by VitD3 or VitD3-Dex (16, 25, 66, 67), which might affect the microenvironment where tolDCs exert their tolerogenic function. Interestingly, tolDCs induced by other agents such as dexamethasone alone or rapamycin did not show similar metabolic changes (68). The increase of metabolic rate through upregulation of oxidative phosphorylation while maintaining or enhancing glycolysis (28, 68), may be a phenomenon similar to the so-called Warburg effect (69). This will result in enhanced glucose uptake and fermentation to lactic acid and may be a target for *in vivo* monitoring upon tolDC treatment.

The ability of tolDC to switch from aerobic respiration to anaerobic glycolysis may have several functional implications. It is presumed to enhance tolDC longevity and resistance to metabolic stress in inflammatory milieu, where low oxygen and glucose levels prevail. Experiments *in vitro* showed that while glucose was essential in the metabolic programming of VitD3 tolDC, the regulatory phenotype remained stable in hypoxic and hypoglycemic conditions after induction (68). The increase in oxidative phosphorylation activity may surge reactive oxygen species (ROS) as byproduct and cause damage to cells with no effective antioxidative machinery in the close proximity (25). The enhanced glucose throughput may cause nutrient deprivation, which can activate intracellular metabolic sensors such as mTOR controlling the homeostatic proliferation of Tregs (70). Thus, metabolic changes inside tolDCs may result in an immune suppressive effect through nutrient deprivation supporting Treg proliferation and the secretion of ROS damaging immune cells in the proximity. It is difficult to envisage how such effects may be monitored *in vivo* as the effect may be local and easily compensated to non-detectable changes in the circulation. Still it may be interesting to explore this uncharted field of immunometabolism as potential functional activity of tolDCs *in vivo*.

## Concluding Remarks

**Figure 1:**
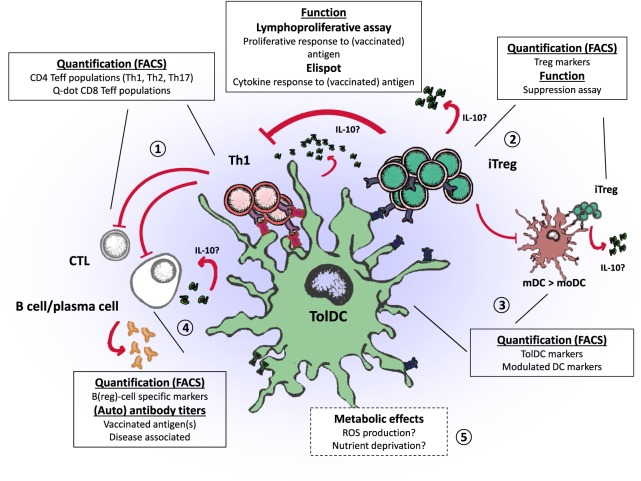
Regulatory properties of tolerogenic dendritic cells (tolDCs) and endpoints for clinical trials. tolDCs: (1) directly inhibit the proliferation of CD4 and CD8 T cells by promoting anergy or apoptosis, (2) prime the induction of regulatory T cells (iTreg) that suppress effector T and B cells, (3) modulate inflammatory dendritic cells (mDC to moDC) through iTregs (infectious tolerance), which in turn can induce regulatory T cells (Tregs) of other specificities through linked suppression, (4) inhibit B cell activity or promote regulatory B cells, and (5) potentially affect immune inflammation through metabolic effects.

Application of tolDC therapy in the clinical setting is an exciting progression toward specific tolerance induction in patients with autoimmune diseases. We now face the challenge to establish the efficacy of tolDC therapy. Results from phase I clinical trials using tolDCs show preliminary effects regarding immune regulation *in vivo*. In this review, we evaluated the regulatory mechanisms of different types of tolDCs to find potential immunological efficacy endpoints, which are summarized in Figure 1. Lymphoproliferative assays measuring the response to disease-associated antigens provide an elegant method to grasp a view of antigen-specific T cell modification, whereas examining affected immune subsets such as Tregs may prove a holy grail that requires appropriate assay improvements. Features that particular tolDCs exert, such as the induction of Bregs may be further explored in other tolDCs, to assess whether these are unique to certain types of tolDCs or common assets. A better understanding of the phenotypical properties of the different tolDC and affected immune cells will provide essential information for choosing the preferred type of tolDC and designing appropriate monitoring endpoint. Therefore, harmonizing assays and following changes in multiple T cell subsets in response to tolDC therapy could enable comparison and correlation to clinical outcomes in different trials.

## Author Contributions

All authors contributed equally to the design and writing of this review.

## Conflict of Interest Statement

The authors declare that the research was conducted in the absence of any commercial or financial relationships that could be construed as a potential conflict of interest. The handling editor declared a past coauthorship with one of the authors TN.
